# Novel broad-spectrum activity-based probes to profile malarial cysteine proteases

**DOI:** 10.1371/journal.pone.0227341

**Published:** 2020-01-10

**Authors:** Michele S. Y. Tan, Dara Davison, Mateo I. Sanchez, Bethany M. Anderson, Stephen Howell, Ambrosius Snijders, Laura E. Edgington-Mitchell, Edgar Deu

**Affiliations:** 1 The Francis Crick Institute, London, United Kingdom; 2 Department of Genetics, Stanford School of Medicine, Stanford, California, United States of America; 3 Department of Biochemistry and Molecular Biology, Bio21 Molecular Science and Biotechnology Institute, The University of Melbourne, Parkville Victoria, Australia; 4 Drug Discovery Biology, Monash Institute of Pharmaceutical Sciences, Monash University, Parkville, Victoria, Australia; 5 Department of Maxillofacial Surgery, College of Dentistry, New York University, New York, New York, United States of America; Institut national de la santé et de la recherche médicale - Institut Cochin, FRANCE

## Abstract

Clan CA cysteine proteases, also known as papain-like proteases, play important roles throughout the malaria parasite life cycle and are therefore potential drug targets to treat this disease and prevent its transmission. In order to study the biological function of these proteases and to chemically validate some of them as viable drug targets, highly specific inhibitors need to be developed. This is especially challenging given the large number of clan CA proteases present in *Plasmodium* species (ten in *Plasmodium falciparum*), and the difficulty of designing selective inhibitors that do not cross-react with other members of the same family. Additionally, any efforts to develop antimalarial drugs targeting these proteases will also have to take into account potential off-target effects against the 11 human cysteine cathepsins. Activity-based protein profiling has been a very useful tool to determine the specificity of inhibitors against all members of an enzyme family. However, current clan CA proteases broad-spectrum activity-based probes either target endopeptidases or dipeptidyl aminopeptidases, but not both subfamilies efficiently. In this study, we present a new series of dipeptydic vinyl sulfone probes containing a free N-terminal tryptophan and a fluorophore at the P1 position that are able to label both subfamilies efficiently, both in *Plasmodium falciparum* and in mammalian cells, thus making them better broad-spectrum activity-based probes. We also show that some of these probes are cell permeable and can therefore be used to determine the specificity of inhibitors in living cells. Interestingly, we show that the choice of fluorophore greatly influences the specificity of the probes as well as their cell permeability.

## Introduction

Malaria remains one of the most devastating infectious diseases worldwide killing close to half a million people and affecting over 200 million every year[1]. Malaria incidence has significantly decreased over the last 15 years mainly due to the distribution of insecticide-impregnated bed nets and the use of artemisinin combination therapy as the standard of care for uncomplicated malaria[2]. However, artemisinin resistance is on the rise[3], and mosquitoes are becoming increasingly resistant to insecticides[4]. Therefore, there is a need to identify and validate new therapeutic targets[5]. Proteases have been pursued as drug targets for a variety of diseases by the pharmaceutical industry due to our structural understanding of their catalytic mechanism and specificity. Indeed, protease inhibitors are currently used to treat hypertension, cancer, diabetes, AIDS, or hepatitis C[6]. Recently, clan CA proteases have gained interest as potential drug targets due to their prominent role in inflammation and cancer[7,8]. Several pharmaceutical companies are currently undertaking drug development programs to treat chronic diseases, such as osteoporosis or bronchiectasis with cathepsin K[9] and cathepsin C (CatC) inhibitors[10–12], respectively. Clan CA protease inhibitors are also being pursued as drugs to treat parasitic diseases such as Chagas disease[13].

In *Plasmodium* species, this family of proteases has been shown to be involved in essential biological process throughout the parasite life cycle[14]. This family is composed of 10 members in *P*. *falciparum* and includes 3 dipeptidyl aminopeptidases (DPAPs) and 7 endopeptidases: 4 falcipains (FPs) and 3 SERAs (serine repeat antigens). In order to better understand the biological functions of these proteases and to chemically evaluate their potential as antimalarial targets, highly specific inhibitors are required. However, given the large number of clan CA proteases present in *P*. *falciparum*, off-target effects within this family is one of the main hurdles to developing specific compounds. In addition, eleven cysteine cathepsins are expressed in humans. Therefore, highly specific inhibitors that do not target host proteases are needed to study the biological function of malarial proteases within the host context, either in animal models of malaria or during liver stages.

Activity-based probes (ABPs) are small molecules that use the enzymatic mechanism of an enzyme to covalently modify its active site[15]. A tag embedded within the structure of the probe, usually a biotin or fluorophore, allows for visualization of the labelled enzymes in a gel-based format. Biotinylated probes can also be used to pull-down the labelled proteins for MS identification. Broad-spectrum ABPs are designed to covalently modify all members of an enzyme family, thus allowing profiling of the activity of each member in a biological sample. This method known as activity-based protein profiling (ABPP) has been broadly applied to determine the specificity of inhibitors given that binding to the active site will prevent probe labelling. Although several ABPs have been developed for clan CA proteases, these probes generally either target endopeptidases or DPAPs, but not both subfamilies. For example, DCG04, which was developed based on the structure of the general covalent cysteine protease inhibitor E64[16], labels most mammalian cysteine cathepsins and the parasite FPs very efficiently, but is very inefficient at labelling *Plasmodium* DPAPs or CatC[17,18]. On the other hand, the DPAP selective probe, FY01, does not label all endopeptidases efficiently[18,19]. Therefore, to profile the specificity of inhibitors against all clan CA proteases two probes are usually required. In this study, we report a new series of broad-spectrum ABPs that is able to label both of these subfamilies.

*Plasmodium* clan CA proteases play important roles throughout the parasite life cycle. Malaria is transmitted through the bite of *Anopheles* mosquitoes. Parasites initially establish an asymptomatic infection in the liver where they multiply into thousands of merozoites (extracellular form that is able to infect erythrocytes). Release of parasites from infected hepatocytes into the blood stream initiates the erythrocytic cycle, which consists of red bood cell (RBC) invasion, parasite growth (ring to trophozoite stage transition), nuclear division and parasite replication (schizogony), and egress from the infected RBC (iRBC). This exponential asexual replication is responsible for the symptoms and pathology of malaria. A fraction of parasites develop into male and female gametocytes, which, after being ingested during a blood meal, will mature into gametes and sexually reproduce in the mosquito midgut. Parasites will then multiply and travel to the salivary glands from where they will be transmitted to the next human host.

Three subfamilies of clan CA proteases are conserved in *Plasmodium* species. In *P*. falciparum the SERA subfamily is composed of 9 members, each containing a papain-like domain, but only 3 have a catalytic cysteine (SERA6-8)[20]. The others have a serine instead and are predicted to be inactive. These proteases are secreted into the parasitophorous vacuole (PV) as zymogens. The PV is a membrane bound vacuole within which parasites develop and replicate isolated from the host cytosol. SERAs are proteolytically activated by a subtilisin-like protease that is released into the PV 15 min before egress, and therefore are predicted to be active for a very short period of time[21,22]. SERA6 has been shown to be essential for parasite egress from iRBCs, and this function is dependent on its catalytic cysteine[22]. Finally, the homologue of SERA8 in *P*. *berghei* has been shown to be essential for parasite egress in insect stages[23].

FPs are endopeptidases active at acidic pH and are expressed during the erythrocytic cycle[24–26]. FP2a, FP2b, and FP3 localize to the digestive vacuole and are at the top of the proteolytic pathway responsible for haemoglobin degradation[14]. This pathway provides a source of amino acids for protein synthesis and liberates space within the iRBC to allow parasite growth. Because FP2a, FP2b, and FP3 perform redundant functions, their individual knockout (KO) does not result in parasite death[27]. However, to the best of our knowledge, a triple KO has not yet been attempted in *P*. *falciparum*. The biological role of the fourth falcipain, FP1, is not well understood. Studies with small molecules suggested a role in RBC invasion[17], but KO of FP1 in *P*. *falciparum* showed no clear phenotype in asexual stages[28]. However, KO of the FP1 homologue in the *P*. *berghei* murine model of malaria impairs invasion of mature RBCs albeit not of reticulocytes[29].

Finally, three DPAPs are conserved in *Plasmodium* species. DPAPs are homologues of CatC and cleave dipeptides off the N-terminus of substrates. Compared to other clan CA proteases, DPAPs have an additional domain, known as the exclusion domain, that interacts with the free N-terminal amine of substrates via its N-terminal Asp side chain. This exclusion domain also prevents binding beyond the P2 position, thus preventing endopeptidase activity. DPAP1 is expressed at all stages of parasite development and has been proposed to play a role during the latest stages of the haemoglobin degradation pathway[30,31]. DPAP2 is only expressed in sexual stages, localizes to apical secretory organelles in gametocytes, and has been shown to play an important role in gamete egress from iRBCs[32,33]. Finally, DPAP3 localizes to novel apical organelles in merozoites, it is secreted immediately before parasite egress, and its activity is required for efficient RBC invasion[34].

In this study we present a new series of fluorescent broad-spectrum ABPs able to label clan CA endopeptidases and DPAPs. These dipeptidic vinyl sulfone probes are able to efficiently target the FPs and DPAPs in *P*. *falciparum*, as well as most mammalian clan CA proteases, both in lysates and in intact cells. These new probes are therefore perfect tools to determine the specificity of inhibitors not only against parasite targets but also against potential off-targets in the host.

## Materials and methods

### Probe synthesis

The synthesis and characterization of W-hPG-VS, *D-*W-hPG-VS, SAK1, and SAK2 have been previously described[34,35]. To synthesize the new probes presented here, the trifluoroacetic (TFA) salt of W-hPG-VS (10 mg, 0.018 mmol) and 0.09 mmol of the azide derivates of the tag (Lumiprobe) were dissolved in an eppendorf tube in 0.9 mL of 1:1 water:DMSO. CuSO_4_ (0.018 mmol, 40 μL of a solution 500 mM) and sodium L-ascorbate (14.2 mg, 0.072 mmol) were added, and the reaction was followed by HPLC-MS until total consumption of the azide derivate. The crude reaction was directly purified by preparative reverse-phase chromatography. The combined fractions were concentrated and freeze-dried, yielding a solid that was identified as the TFA salt of the desired product. The LCMS traces of the high-resolution mass for each probe are shown in S1 Fig.

### Parasite culture and lysates preparation

Anonymized human blood to culture malaria parasites was purchased from the United Kingdom National Health System Blood and Transplant Special Health Authority. No ethical approval is required for its use. *Plasmodium falciparum* 3D7 parasites were grown at 2% hematocryt in RPMI 1640 (Gibco) media supplemented with Albumax (Invitrogen) as previously described[35]. Parasite cultures were synchronised by purifying mature schizonts by sedimentation using a Percoll gradient, and incubating them under shaking conditions for 2–3 h with fresh RBCs and media to allow parasite egress and RBC invasion. Unruptured schizonts were removed from the culture using a Percoll gradient, and any remaining schizonts in our ring-stage culture were removed by sorbitol treatment. Mature schizonts were obtained by culturing purified schizonts for 3 h in the presence of 1 μM of the cGMP-dependent protein kinase inhibitor Compound 2[21].

Trophozoite stage parasite pellets were collected 24 h after synchronization, and schizonts were purified using a Percoll gradient 44 h after synchronization. For both, the RBC and PV membranes were lysed using saponin, and the parasite pellets washed multiple times with PBS. Merozoites were purified as previously described[35]. Briefly, purified schizonts were allow to egress in media under shaking conditions. Free merozoites were separated from unruptured schizonts by centrifugation, and the merozoite-containing supernatant passed through a magnet (SuperMACS) to remove any leftover schizonts and hemozoin-containing residual bodies of ruptured iRBCs. All parasite pellets were snap frozen in liquid nitrogen and stored at -80°C. To obtained lysates, parasite pellets were incubated on ice for 1 h in two volumes of 1% NP40 in PBS followed by a 5 min centrifugation at 17,000 x g to obtain the soluble fraction of the lysate.

### Parasite labelling conditions

Parasite lysates were diluted 10-fold in acetate buffer (50 mM sodium acetate, 100 mM NaCl, 5 mM MgCl_2_, and 5 mM DTT, pH 5.5) and treated with different concentrations of probes for 1 h at RT. For competition assays, lysates were pre-treated for 30 min with inhibitor prior to probe labelling. Labelling was stopped by adding 4X loading buffer and boiling the samples for 5 min. Labelling of cysteine proteases in live parasites was performed by diluting purified mature schizonts 10-fold in RPMI containing 1 μM of Compound 2, and treating the samples for 1 h with increasing concentrations of probes at 37°C. The reaction was stopped by added 4X loading buffer and boiling the samples for 10 min. All samples were loaded on a 12% SDS-PAGE gel, and the in-gel fluorescence measured on a PharosFX fluorescence scanner (Biorad) either using a 532 nm laser for probes containing a Cy3, sCy3 or TAMRA (605 nm emission filter), and a 635 nm laser for those with a Cy5 or sCy5 fluorophore (695 nm emission filter).

### RAW macrophage culture, lysates preparation, and labelling

RAW264.7 cells (immortalized murine macrophages) were cultured in DMEM containing 10% fetal bovine serum and 1% antibiotic/antimycotic. Cells were passaged by scraping with a rubber policeman. Cells were plated in 12-well plates (300,000 cells/per well) and allowed to adhere overnight. The indicated probes were added at a final concentration of 1 μM for 3 h. Cells were lysed in 30 μl citrate buffer (50 mM citrate, 0.5% CHAPS, 0.1% Triton X-100, and 4 mM DTT, pH 5.5). Lysates were centrifuged, and cleared supernatants were solubilized with 7.5 μl 5x sample buffer (200 mM Tris-Cl, 8% SDS, 0.04% bromophenol blue, 5% β-mercaptoethanol, and 40% glycerol, pH 6.8). Samples (20 μl) were then resolved on a 15% SDS-PAGE gel, and labeling was detected by scanning for Cy5 or Cy3 fluorescence on a Typhoon 5 flatbed laser scanner (GE Healthcare). For lysate labeling, cells were first lysed as above in citrate buffer and 20 μl lysate was incubated with 1 μM probe for 20 min at 37C°. Sample buffer was added to stop the reaction, and samples were analyzed as above. All samples were run as duplicates.

### Chemical proteomics

Schizont pellets were resuspended in one volume of 0.1% Triton X-100 in PBS buffer, incubated at 4°C for 20 min, and the soluble fraction of the lysate collected after centrifugation at 17,000 x g for 5 min. Protein concentration was determined using a NanoDrop 2000 spectrometer (Thermo Scientific). Lysates were diluted in acetate buffer to a protein concentration of 2 mg/mL, and six 100 μl samples were treated for 10 min either with DMSO or 1 μM W-BF-VS. Protein was then precipitated by adding 200 μL of methanol, 50 μL of chloroform and 100 μL of water, and the pellet collected by centrifugation (2 min at 17,000 x g). Protein was first resolubilized in 60 μL of 2% SDS and 10 mM DTT in PBS, and diluted 10-fold in PBS to achieve a concentration of 1 mg/mL. A mixture of 10 μL of neutravidin-agarose beads and 20 μL of blank agarose beads (both from Pierce) was added to the samples and incubated at RT for 2 h under shaking conditions. Beads were then collected by centrifugation (2 min at 3000 x g) and washed three times with 1% SDS in PBS, two times with 4 M urea in PBS, and two times with 50 mM ammonium bicarbonate (AMBIC). Beads were then treated with 10 mM tris(2-carboxyethyl)phosphine hydrochloride in 50 mM AMBIC for 30 min at RT, washed, resuspended in 30 μL of 50 mM AMBIC, and treated with 0.12 μg of trypsin gold (Promega) overnight at 37°C under shaking conditions. After collecting the supernatant, peptides were further eluted with 70 μL of 1.5% TFA.

The combined peptide mixture was filtered and desalted on homemade ‘stage tips’ containing C18 Empore membrane (Empore Octadecyl C18 47mm Extraction Disks 2215 Supleco 66883-U). Tips were washed by centrifugation (2 min at 2000 x g) with 150 μL of MeOH and 150 μL of water. The peptide sample (140 μl) was loaded, the tips washed with 150 μL of water, and eluted with 60 μl of 40% acetonitrile in water. The peptide solution was then evaporated to dryness using a speed vacuum (SpeedVac system ISS 100, Savant Integrated). Peptide samples were dissolved in 25 μL of 50 mM triethylammonium bicarbonate buffer (Sigma), and 20 μl of the appropriate TMT label (ThermoFisher TMTsixplex product number 90308, lot number RH239931) freshly dissolved in anhydrous acetonitrile was added to each of the six samples and incubated for 1 h at RT. Combined TMT samples were then fractioned into 8 samples using a high pH reversed-phase peptide fractionation kit (Pierce 84868). Fractionated samples were then evaporated to dryness using a speed vacuum.

Peptides were chromatographically resolved on an Ultimate 3000 nanoRSLC HPLC (Thermo Scientific). Each peptide fraction was acidified to a final concentration of 0.1% TFA, and 1–10 μl loaded onto a 2 x 0.3 mm Acclaim Pepmap C18 trap column (Thermo Scientific) at 15 μl/min of 0.1% TFA prior to the trap being switched to elute at 0.25 μl/min through a 50 cm x 75 μm EasySpray C18 column. In a 90 min run, the following gradients of solution A (2% acetonitrile, 0.1% formic acid) and B (80% acetonitrile, 0.1% formic acid) were used: 9–25% solution B over 37 min, 25–40% solution B over 18 min, 100% solution B over 15 min, and equilibration back to 2% solution B for 20 min. HPLC eluant was introduced into an Orbitrap Fusion Lumos (Thermo Scientific). The Orbitrap was operated in “Data Dependent Acquisition” mode with a survey scan at a resolution of 120,000 from m/z 400–1400. This was followed by “TopS” precursor ion selection MS/MS using 38% high energy collision dissociation. Dynamic exclusion was used with a time window of 30 s.

### Proteomics data analysis

The raw data files were analysed using MaxQuant (version 1.6.2.1.). Quantification was done at MS2 level using 6-plex TMT labels, no label free quantification was performed. Default MaxQuant settings were used with the *Homo sapiens* (Uniprot 13/01/2013) and *Plasmodium falciparum* 3D7 (PlasmoDB 15/12/2016) sequence databases. A decoy database of reversed sequences was used to filter false positives at a peptide false detection rate of 1%.

The data files generated by MaxQuant were further analyzed using Perseus (version 1.5.6.0.). The list of proteins identified by MaxQuant was filtered to remove proteins that were only identified by a modification site, proteins identified from the reverse decoy peptide database, and proteins annotated as potential contaminants. Proteins that were identified from 2 peptides or less were also excluded from the analysis. After log2(x) transformation of the TMT reporter ion intensities, the data was normalised by subtracting the median intensity from each channel to correct for differences in loading. Two sample t-tests with multiple hypothesis testing correction (S0 = 1, permutation based FDR = 0.05 using default Perseus settings) were carried out between DMSO and probe treated samples in order to identify statistically significant differences. The results were visualised on a “volcano plot” (S2 Fig).

## Results and discussion

### Probe design

**Fig 1:**
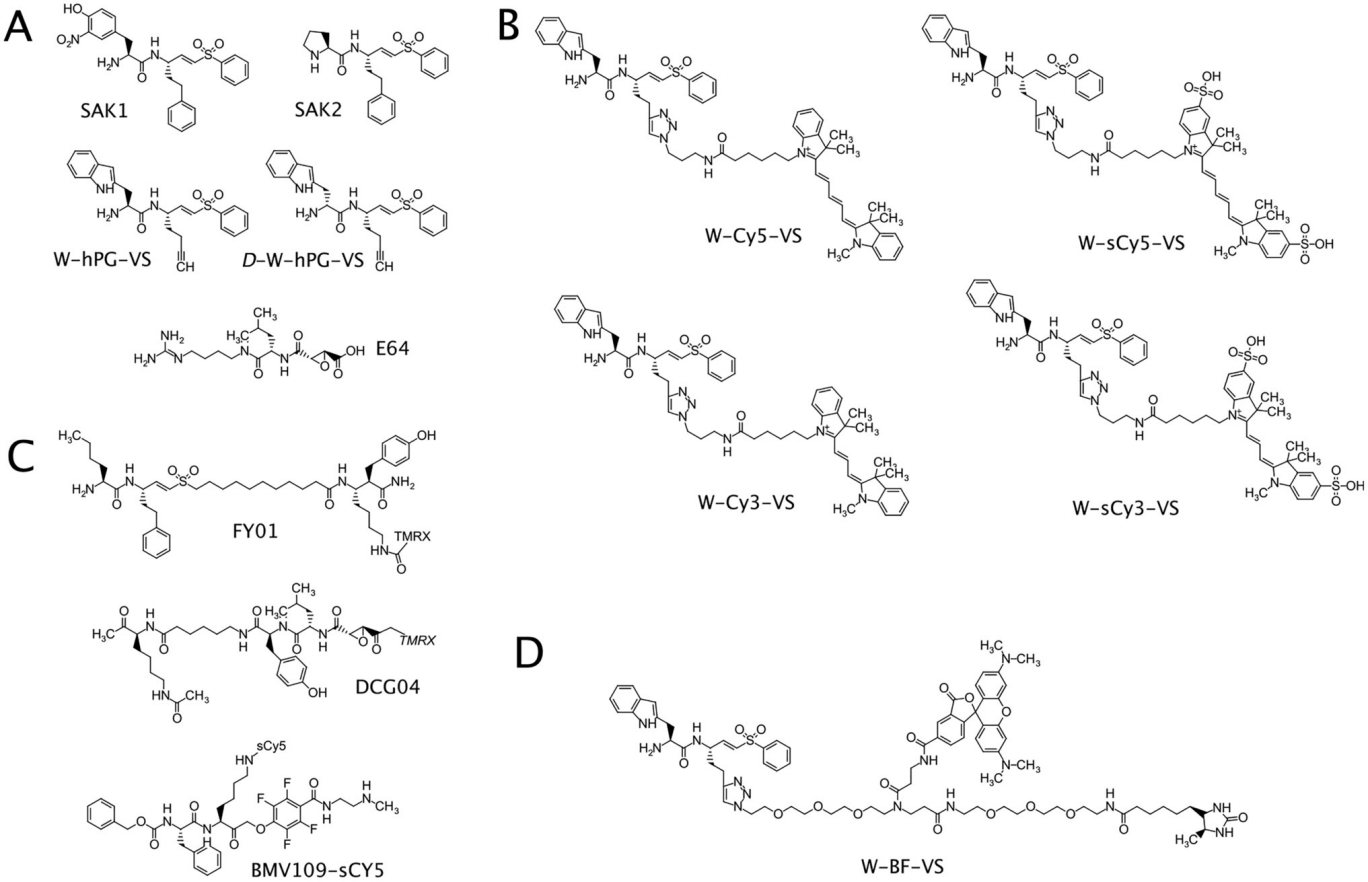
Structures of inhibitors and ABPs. (**A**) Structures of inhibitors used in this study. All the new ABPs described in this paper were synthesized by conjugating different azido-tags (**B**, fluorophore or **D**, a biotin/TAMRA bifunctional tag) to the alkyne group of W-hPG-VS. (**C**) Structures of previously published ABPs used in this study for comparison purposes.

Our initial intention was to develop highly selective DPAP3 probes based on the observation that dipeptidic vinyl sulfone inhibitors with a free N-terminal Trp are DPAP3 specific[34,36]. Because the S1 pocket is generally solvent exposed in clan CA proteases, we decided to introduce an alkyne group at the P1 position and use copper-catalyzed click chemistry to conjugate different azido-tagged fluorophores. We therefore synthesize the precursor inhibitor Trp-hPG-VS (Fig 1A) and conjugated different fluorophores (Cy5, sulfoCy5, Cy3, or sulfoCy3) to the homoprolylglycine (hPG) side chain (Fig 1B). We named these probes W-Cy5-VS, W-sCy5-VS, W-Cy3-VS, and W-sCy3-VS, respectively. We then determined the specificity of these probes in parasite lysates and compared them to ABPs previously used to label malarial cysteine proteases, namely FY01 and DCG04 (Fig 1C).

### Probe specificity in parasite lysates

**Fig 2:**
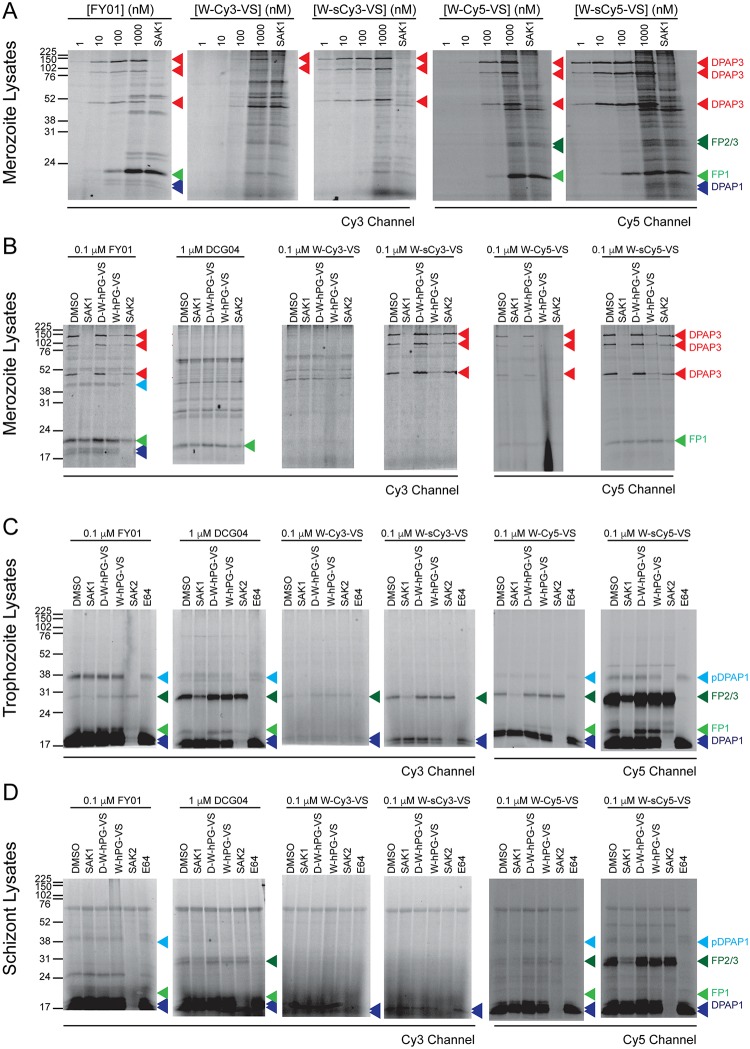
Labelling of cysteine proteases in parasite lysates. (**A**) Merozoite lysates diluted 1:10 in acetate buffer were treated for 1 h with 1–1000 nM of the indicated ABPs. For the highest ABP concentration, samples were also pre-treated for 30 min with 1 μM of the DPAP3 inhibitor SAK1, which results in the loss of labelling of the three isoforms of DPAP3 running at 120, 95, and 42 kDa. (**B-D**) Lysates collected at merozoite (**B**), trophozoite (**C**), or schizont (**D**) stages were diluted in acetate buffer (pH 5.5), pre-treated for 30 min with DMSO or 10 μM of different known covalent inhibitors of DPAP1 (SAK2), DPAP3 (SAK1 or W-hPG-VS), the FPs (E64), or the negative control compound *D*-W-hPG-VS. This was followed by 1 h labelling with the different ABPs at 0.1 μM except for DCG04 that was used at 1 μM concentration. (**A-D**) The fluorescent bands corresponding to DPAP1, DPAP3, FP1, and FP2/3 are indicated by blue, red, light green, and dark green arrowheads, respectively. Two additional biological replicates of these experiments are shown in S3 Fig.

We first tested our probes in merozoite lysates since DPAP3 is most abundant at this stage. Lysates were treated for 1 h with a probe concentration ranging from 1 nM to 1 μM. Samples were then run on a SDS-PAGE gel, and the labelled proteins visualized using a fluorescence scanner. At the highest probe concentration lysates were also pre-treated with the DPAP3-selective covalent inhibitor SAK1[18,36] to confirm whether the probes labelled DPAP3 (Fig 1A). In merozoite lysates under acidic conditions FY01 has been shown to label three different forms of DPAP3 running at 120, 95, and 42 kDa[18]. As shown in Fig 2A, all probes are able to label the three forms efficiently with the exception of W-Cy3-VS. W-sCy5-VS and W-sCy3-VS are able to label DPAP3 at 1 nM, and the latter is DPAP3-selective when used between 1 and 100 nM. However, all probes start labelling other targets between 0.1 and 1 μM. In particular, W-sCy5-VS labels a large number of proteins, making it a potential broad-spectrum ABP.

To further determine the specificity of these probes, we tested them at 100 nM in parasite lysates collected at different stages of development (merozoites, trophozoites and schizonts). To determine whether these probes target other known clan CA proteases, lysates were pre-treated with covalent inhibitors of DPAP1 (SAK2), DPAP3 (SAK1), or the FPs (E64)[18]. We also included the W-hPG-VS, compound from which the probes were derived, in this competition assay, as well as the diastereomer control *D-*W-hPG-VS (Fig 1A) that does not inhibit DPAP3 or any other clan CA protease[36]. Despite being DPAP3-selective in merozoite lysates (Fig 2A and 2B), most probes also target DPAP1 and the FPs in trophozoite and schizont lysates (Fig 2C and 2D), probably because these other proteases are much more abundant at these stages than in merozoites. W-sCy5-VS is able to very robustly label all DPAPs and FPs, as well as the pro-form of DPAP1 running at 38 kDa (Fig 2C and 2D). Although W-Cy5-VS also has broad specificity, it is a much weaker probe than W-sCy5-VS. Surprisingly, the probes containing the Cy3 and sCy3 fluorophores show very poor labelling of cysteine proteases under these conditions. These results illustrate how the choice of fluorophore can have a major influence on the potency and specificity of probes.

As reported previously, the W-hPG-VS inhibitor is a specific inhibitor of DPAP3[36], but conjugating a fluorophore to the P1 position via cupper-catalyzed click chemistry results in a very significant loss of specificity, especially with sCy5. This was surprising as the P1 position is generally solvent exposed in clan CA proteases. However, substrate specificity studies on DPAPs have shown that long and hydrophobic non-natural amino acids at the P1 position greatly increase substrate turnover, possibly allowing the P1 residue to interact with an adjacent hydrophobic pocket[35]. This might also be the case for the FPs and might explain why our probes are no longer specific for DPAP3 when compared to W-hPG-VS. Another potential explanation is that the fluorophore might allow the probe to make non-specific hydrophobic interactions, increasing the time available for the catalytic cysteine to attack the vinyl sulfone warhead. All labelling experiments in parasite lysates were performed in triplicate, the two replicates not shown in Fig 2 are shown in S3 Fig.

### Probe specificity in live parasites

**Fig 3:**
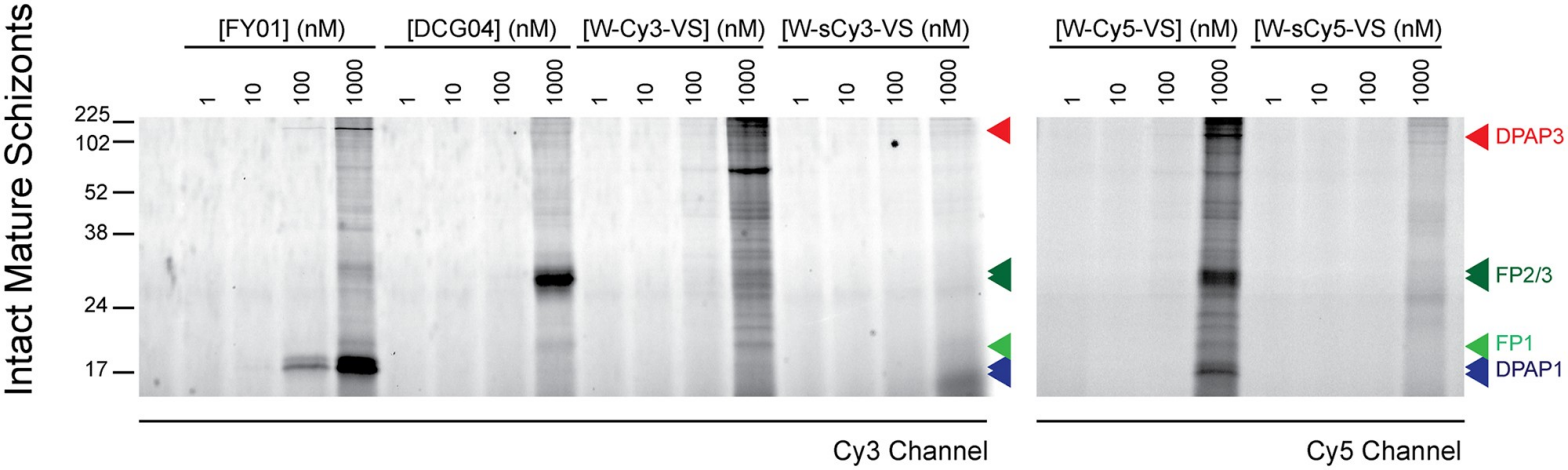
Labelling of cysteine protease in live parasites. Very mature schizonts were diluted ten-fold in RPMI and treated with different concentrations of probes for 1 h. Samples were run on a SDS-PAGE gel, and the labelled proteins detected using at fluorescence scanner. Bands corresponding to DPAP1, DPAP3, FP1, and FP2/3 are indicated by blue, red, light green, and dark green arrowheads, respectively. Two additional biological replicates of this experiment are shown in S4 Fig.

We then tested the specificity of these probes in live parasites by treating very mature schizonts for 1 h with different concentrations of probes. Purified schizonts were arrested 15 min before egress by treating them with an inhibitor of the cGMP-dependent protein kinase[21]. We used these labelling conditions because DPAPs and FPs are active and abundant at this stage, and because the RBC and PV membranes are still intact, thus allowing us to determine the cell-permeability of our probes. For all probes, clear labelling of cysteine proteases only occurs at 1 μM (Fig 3). It is also quite clear that the sulfate groups in W-sCy3-VS and W-sCy5-VS prevent the probes from crossing membranes and reaching their targets. Under these conditions, DCG04 only labels the FPs, and FY01 the DPAPs. However, W-Cy5-VS is able to label all of these proteases, thus making it a very useful broad-spectrum ABP that can be used in live parasites. Note that only the p120 form of DPAP3 is labelled in live parasites. This is consistent with our previous results showing that DPAP3 processing to the p95 and p42 form is an artefact of parasite lysis, and that the only form found in live parasites is the p120 form[34]. Intact labelling experiments were performed in triplicate, the two replicates not shown in Fig 3 are shown in S4 Fig.

### Confirmation of probe selectivity through chemical proteomics

**Fig 4:**
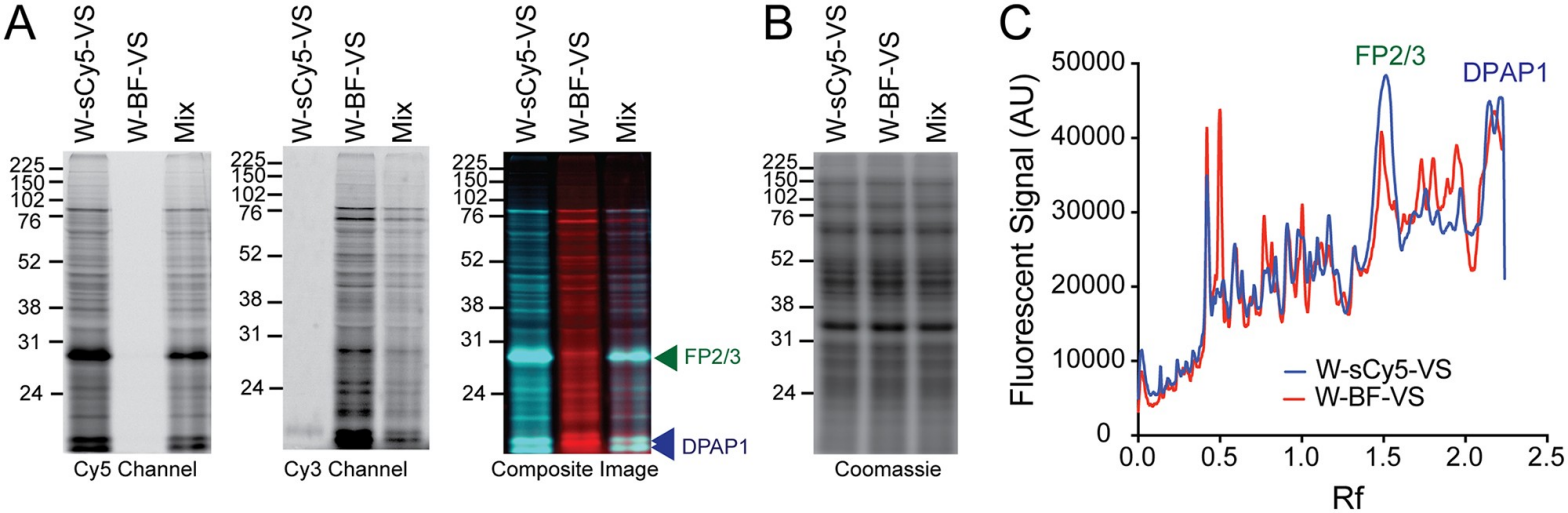
Comparison of labelling profiles of W-sCy5-VS and W-BF-VS. (**A**) Schizont lysates were treated for 1 h either with 0.5 μM of W-sCy5-VS, W-BF-VS, or a mixture of both probes, each at 0.5 μM (Mix). After running the samples in a SDS-PAGE gel, the gel was scanned either in the Cy5 and Cy3 channels. The composite image shows very similar labelling profiles for both probes and a clear co-migration of the labelled bands in the Mix sample. (**B**) Coomassie staining of the gel shown in **A** showing equal protein loading. (**C**) Quantification of the labelling profiles for each probe by densitometry. Fluorescent intensity vs. migration distance (Rf) is shown. The position of FP2/3 and DPAP1 are indicated in A and C. Two additional biological replicates of this experiment are shown in S5 Fig.

To better determine the specificity of these probes in parasite lysates, we synthesized a probe with a bifunctional tag containing a TAMRA fluorophore and a desthiobiotin moiety for pull-down and MS ID purposes (W-BF-VS, Fig 1D). Since W-sCy5-VS is the probe with broader specificity in parasite lysates, we compared its labelling profile to that of W-BF-VS in schizont lysates at 0.5 μM. We also tried a competition labelling experiment where both probes were added to the lysate simultaneously. Most proteins labelled by both probes run at the same MW (Fig 4A). However, as the MW decreases, the TAMRA-labelled proteins run at a slightly higher MW, which is consistent with W-BF-VS being a much larger probe than W-sCy5-VS (Fig 1). This is very evident when comparing the labelling of the DPAP1 doublet. Quantification of these labelling patterns by densitometry clearly show very similar profiles for both probes (Fig 4C). In addition, for most of the bands we observed a clear decrease in fluorescence intensity in both fluorescence channels when the probes were competed against each other. Thus, both these probes seem to have very similar specificities. Labelling experiments comparing W-sCy5-VS and W-BF-VS were performed in triplicate. Those not showin in Fig 4 are shown in S5 Fig.

We then used the W-BF-VS probe to affinity-purify its targets and identify them by MS. Briefly, schizont lysates were treated with DMSO or W-BF-VS under acidic conditions, the modified proteins were then pulled-down with streptavidin agarose beads and after multiple stringent washes, trypsin was added to the beads. This was performed in triplicate, and the tryptic peptides from each sample labelled with a different tandem mass tag (TMT). TMT-labelled peptides were pooled and fractionated into 8 samples by high pH reversed-phase fractionation. Peptides in each fraction were detected and quantified by LCMS/MS/MS analysis.

Table 1 shows the 10 most enriched proteins (Table 1 and S1 Fig) when comparing probe vs DMSO treatment. Among these, we could identify FP2, FP3 and both DPAPs, but not FP1. Note that FP2a and FP2b only differ in a single amino acid, therefore, the peptides identified for these proteins could not be assigned to either one of them. Interestingly, among the most enriched proteins we also identified several enzymes involved in redox catalysis such as thioredoxin or a disulphide isomerase, which are known to contain highly reactive catalytic cysteines. These might react with the vinyl sulfone group in our probes. Therefore, some of the unidentified bands labelled by W-sCy5-VS or W-BF-VS might correspond to proteins containing highly nucleophilic cysteines. Overall, this chemical proteomic experiment confirms that the main targets of our probes are indeed clan CA proteases.

**Table 1:** Top ten enriched proteins using the W-BF-VS probe.

	Gene ID	Protein Name	Fold-Enrichment	Comments
1	PF3D7_1116700	**DPAP1**	8.9	Cysteine Protease
2	PF3D7_0827900	*PDI8*	6.4	Protein disulphide isomerase
3	PF3D7_1115400	**FP3**	4.5	Cysteine Protease
4	PF3D7_1115300/700	**FP2a/b**	3.9	Cysteine Protease
5	PF3D7_1205600	Tetratricopeptide repeat protein	3.8	Putative protein, unknown function
6	PF3D7_1302100	G27/25	3.3	Gamete antigen 27/25
7	PF3D7_1352500	*Thioredoxin-related protein*	3.3	Putative thioredoxin
8	PF3D7_1108600	ERC	3.1	ER-resident calcium binding protein
9	PF3D7_0404700	**DPAP3**	3.0	Cysteine Protease
10	PF3D7_0622800	Leu-tRNA ligase	2.8	Putative protein

Clan CA proteases are indicated in bold, redox enzymes containing a catalytic cysteine in italic.

### Probe specificity in mammalian cells

**Fig 5:**
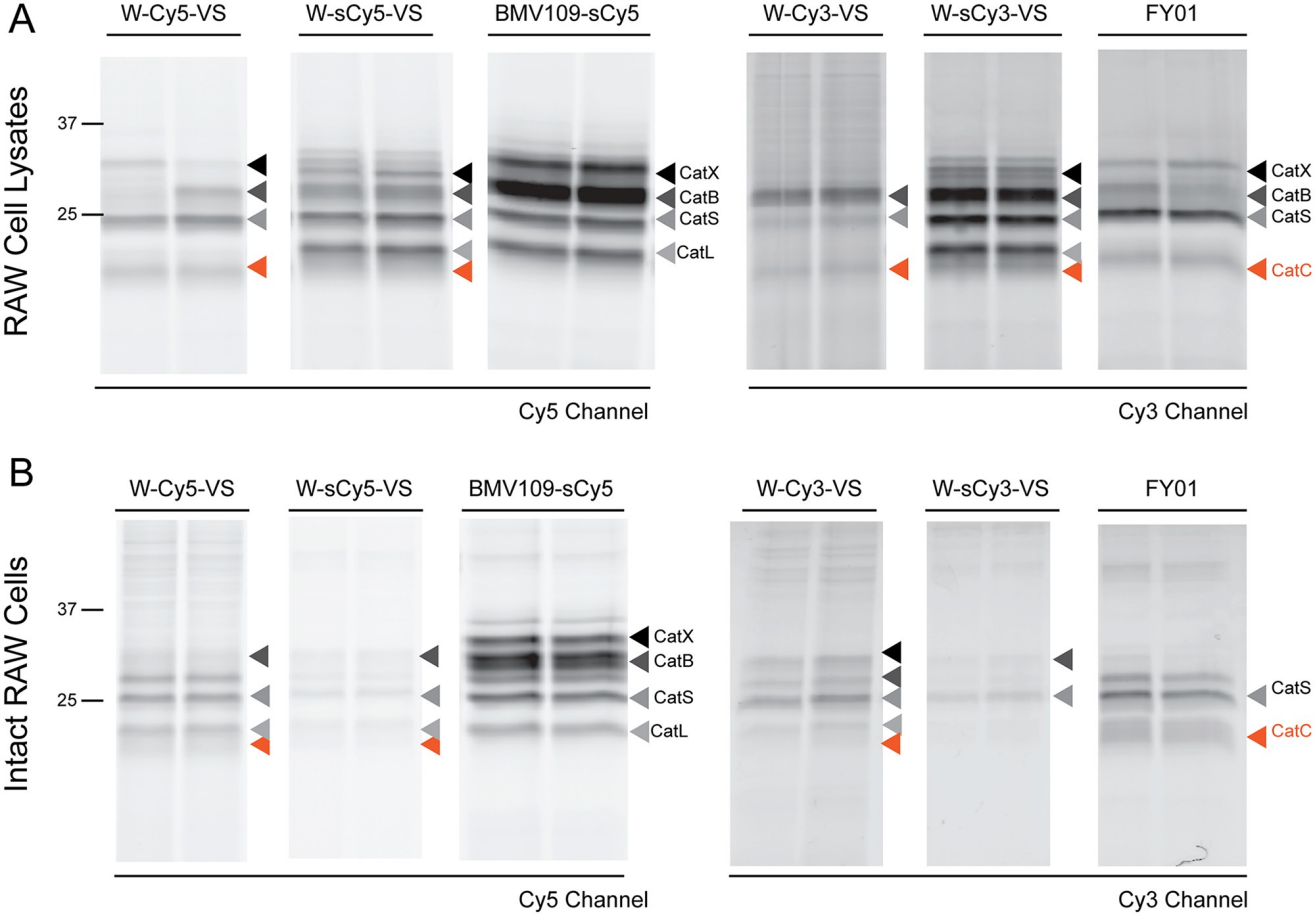
Labelling of cysteine cathepsins in mammalian cells. (**A**) Lysates from RAW macrophages were treated with 1 μM of the indicated ABPs for 30 min. (**B**) Live RAW cells were treated with 1 μM of the indicated ABPs for 3 h. Samples were run on a SDS-PAGE gel, and in-gel fluorescence measured using a fluorescence scanner. The identity of the different cysteine cathepsins are indicated with different coloured arrowheads.

Finally, to determine whether our probes, especially W-sCy5-VS and W-Cy5-VS, could also be used to profile mammalian clan CA proteases, we tested them in RAW macrophages, both in lysates and intact cells (Fig 5). The labelling profiles of these probes were compared to that of FY01 and BMV109-sCy5, a non-peptidic quenched ABP that has been shown to efficiently label most cysteine cathepsin endopeptidases in mammalian cells[37]. As shown in Fig 5A, both FY01 and our probes label a diffuse band running below the CatL band that corresponds to CatC[19]. This band is more clearly observable in the W-Cy5-VS and FY01 treated samples because these two probes do not label CatL. W-sCy3-VS and W-sCy5-VS are able to label all the cysteine cathepsins labelled by BMV109 in addition to CatC. In intact macrophages, W-Cy5-VS is able to label most clan CA endopeptidases but not as efficiently as BMV109. However, contrary to BMV109, W-Cy5-VS is also able to label CatC in intact cells. Here, we also observed that probes having sCy3 and sCy5 label much less efficiently that those containing Cy3 or Cy5, indicating that negatively charged fluorophores impair cell permeability. Similar results were obtained when this probes were tested in mouse lung and spleen tissues, or in DC1940 mouse dendritic cells and SSC-9 human oral cancer cells (S6 Fig), thus showing that W-Cy5-VS and W-sCy5-VS are able to label most clan CA cysteine proteases in a variety of mammalian samples.

## Conclusion

Overall, we have shown that W-sCy5-VS is a broad-spectrum clan CA proteases ABP able to efficiently label endopeptidases and DPAPs both in *P*. *falciparum* and mammalian cell lysates, thus making it a better probe to profile this enzyme family than currently available ones. Unfortunately, the negatively charge nature of the sCy5 fluorophore significantly reduces its cell permeability. However, replacement of the sCy5 with Cy5 allows the probe to cross membranes, thus making W-Cy5-VS an ideal tool to profile the activity of these proteases in living cells.

## Supporting information

S1 Fig Probes purity. LCMS traces (left) of the different ABPs synthesized for this study and accurate mass spectra (right) detected at the maximum peak of absorbance.
(PDF)

S2 Fig Enrichment of proteins labelled with the W-BF-VS affinity probe. Volcano plot showing enrichment of clan CA proteases and enzymes containing reactive cysteine when schizont lysates were treated with W-BF-VS versus DMSO.
(TIFF)

S3 Fig Labelling of cysteine proteases in parasite lysates, additional biological replicates. (**A**) Merozoite lysates diluted 1:10 in acetate buffer were treated for 1 h with 1–1000 nM of the indicated ABPs. For the highest ABP concentration, samples were also pre-treated for 30 min with 10 μM of SAK1, W-hPG-VS *D-*W-hPG-VS, SAK2 or E64. (**B-C**) Lysates collected at trophozoite (**B**) or schizont (**C**) stages were diluted in acetate buffer (pH 5.5), pre-treated for 30 min with DMSO or 10 μM of different known covalent inhibitors of DPAP1 (SAK2), DPAP3 (SAK1 or W-hPG-VS), the FPs (E64), or the negative control compound *D*-W-hPG-VS. This was followed by 1 h labelling with the different ABPs at 0.1 μM except for DCG04 that was used at 1 μM concentration. (**A-C**) The fluorescent bands corresponding to DPAP1, DPAP3, FP1, and FP2/3 are indicated by blue, red, light green, and dark green arrowheads, respectively. Each page represent a different biological replicate.
(PDF)

S4 Fig Labelling of cysteine protease in live parasites, additional biological replicates. Very mature schizonts were diluted ten-fold in RPMI and treated with different concentrations of probes for 1 h. Samples were run on a SDS-PAGE gel, and the labelled proteins detected using at fluorescence scanner. Bands corresponding to DPAP1, DPAP3, FP1, and FP2/3 are indicated by blue, red, light green, and dark green arrowheads, respectively. Top and bottom panels represent different biological replicates.
(TIF)

S5 Fig Comparison of labelling profiles of W-sCy5-VS and W-BF-VS, additional biological replicates. (**A**) Schizont lysates were treated for 1 h either with 0.5 μM of W-sCy5-VS, W-BF-VS, or a mixture of both probes, each at 0.5 μM (Mix). (**B**) Schizont lysates were pre-treated for 30 min with either 10 μM of E64 or 0.5 μM of one of the probes followed by 1h labelling with the other probe or a combination of both, each at 0.5 μM (last two lanes). After running the samples in a SDS-PAGE gel, the gel was scanned either in the Cy5 and Cy3 channels. The composite image (merge channels images) shows very similar labelling profiles for both probes and a clear co-migration of the labelled bands in the Mix sample. Note that E64 is able to outcompete probe labelling for most of the bands. While pre-treatment with W-sCy5-VS results in a decrease in the labelling intensity of the bands labelled by W-BF-VS, pre-treatment with W-BF-VS does not decrease the labelling of band by W-sCy5-VS. This is consistent with W-sCy5-VS being a more potent and reactive probe.
(TIF)

S6 Fig Labelling of mammalian cysteine cathepsins, additional experiments. Lysates from the indicated cell lines or from mouse lung or spleen tissues were treated with 1 μM of the different ABPs for 30 min. The identity of the different cysteine cathepsins are indicated with different coloured arrowheads.
(TIF)
