# Correction: Novel broad-spectrum activity-based probes to profile malarial cysteine proteases

**DOI:** 10.1371/journal.pone.0231231

**Published:** 2020-03-26

**Authors:** Michele S. Y. Tan, Dara Davison, Mateo I. Sanchez, Bethany M. Anderson, Stephen Howell, Ambrosius P. Snijders, Laura E. Edgington-Mitchell, Edgar Deu

The sixth author's name is missing a middle initial. The correct name is: Ambrosius P. Snijders. The correct citation is: Tan MSY, Davison D, Sanchez MI, Anderson BM, Howell S, Snijders AP, et al. (2020) Novel broad-spectrum activity-based probes to profile malarial cysteine proteases. PLoS ONE 15(1): e0227341. https://doi.org/10.1371/journal.pone.0227341
