# Supplementary figures and images for: Novel broad-spectrum activity-based probes to profile malarial cysteine proteases
Source: PLoS One. 2020 Jan 10;15(1):e0227341. doi: 10.1371/journal.pone.0227341 (PMC6953825; doi:10.1371/journal.pone.0227341)

LCMS Trace

m/z Ions

W-Cy5-VS

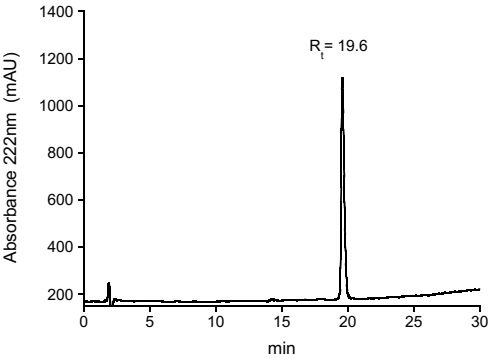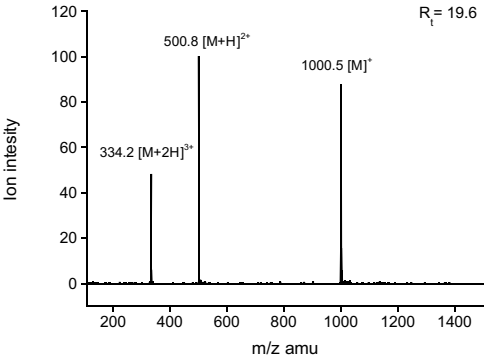

W-sCy5-VS

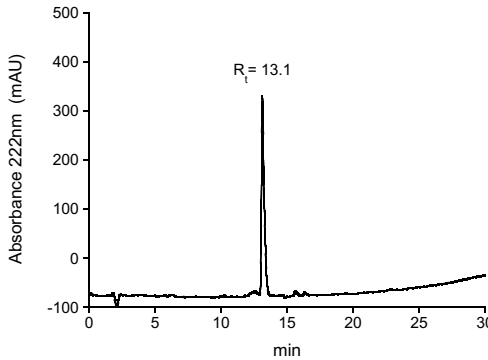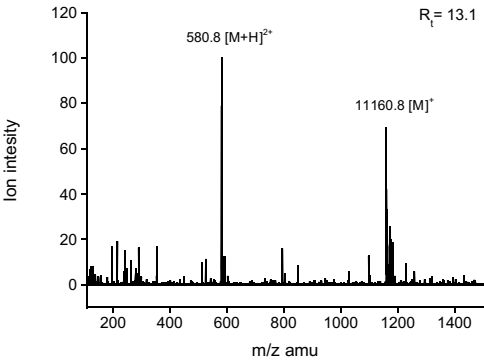

W-Cy3-VS

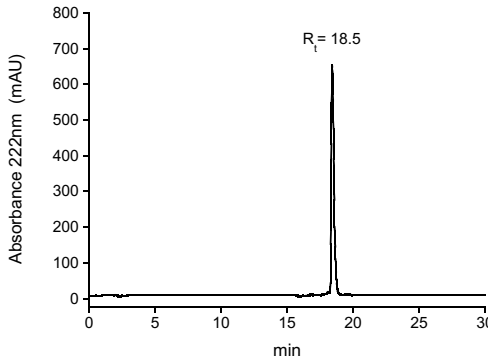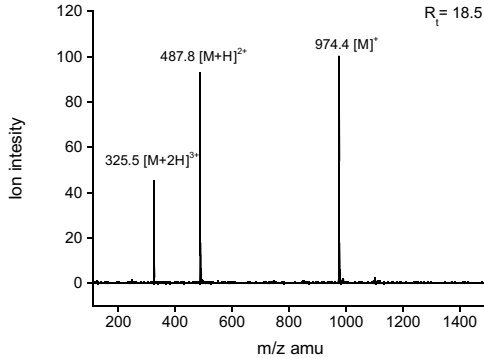

W-sCy3-VS

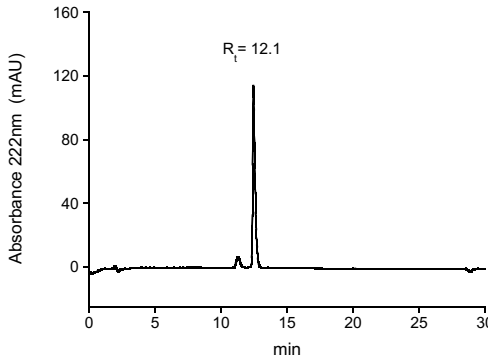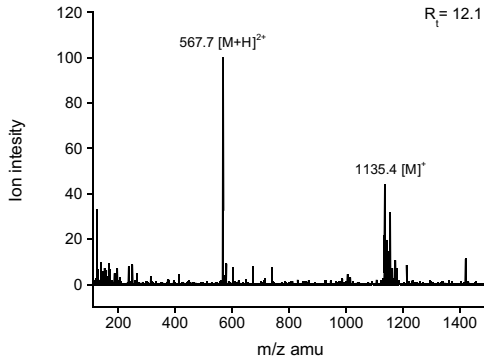

W-BF-VS

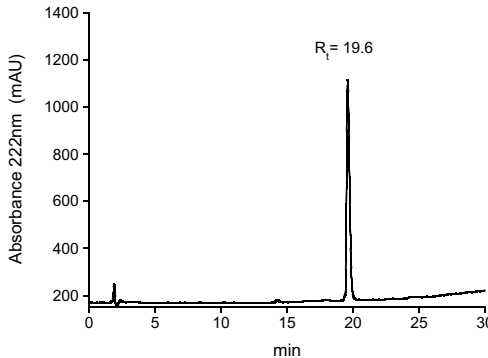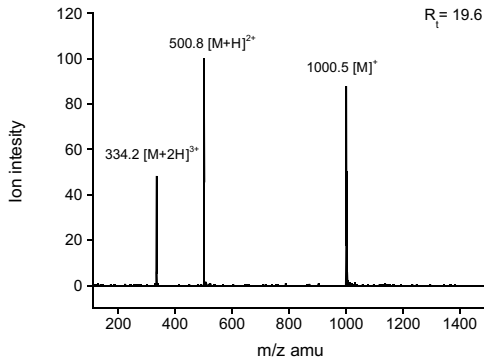

Supplement: S1 Fig — LCMS traces (left) of the different ABPs synthesized for this study and accurate mass spectra (right) detected at the maximum peak of absorbance. (PDF) [file pone.0227341.s001.pdf]

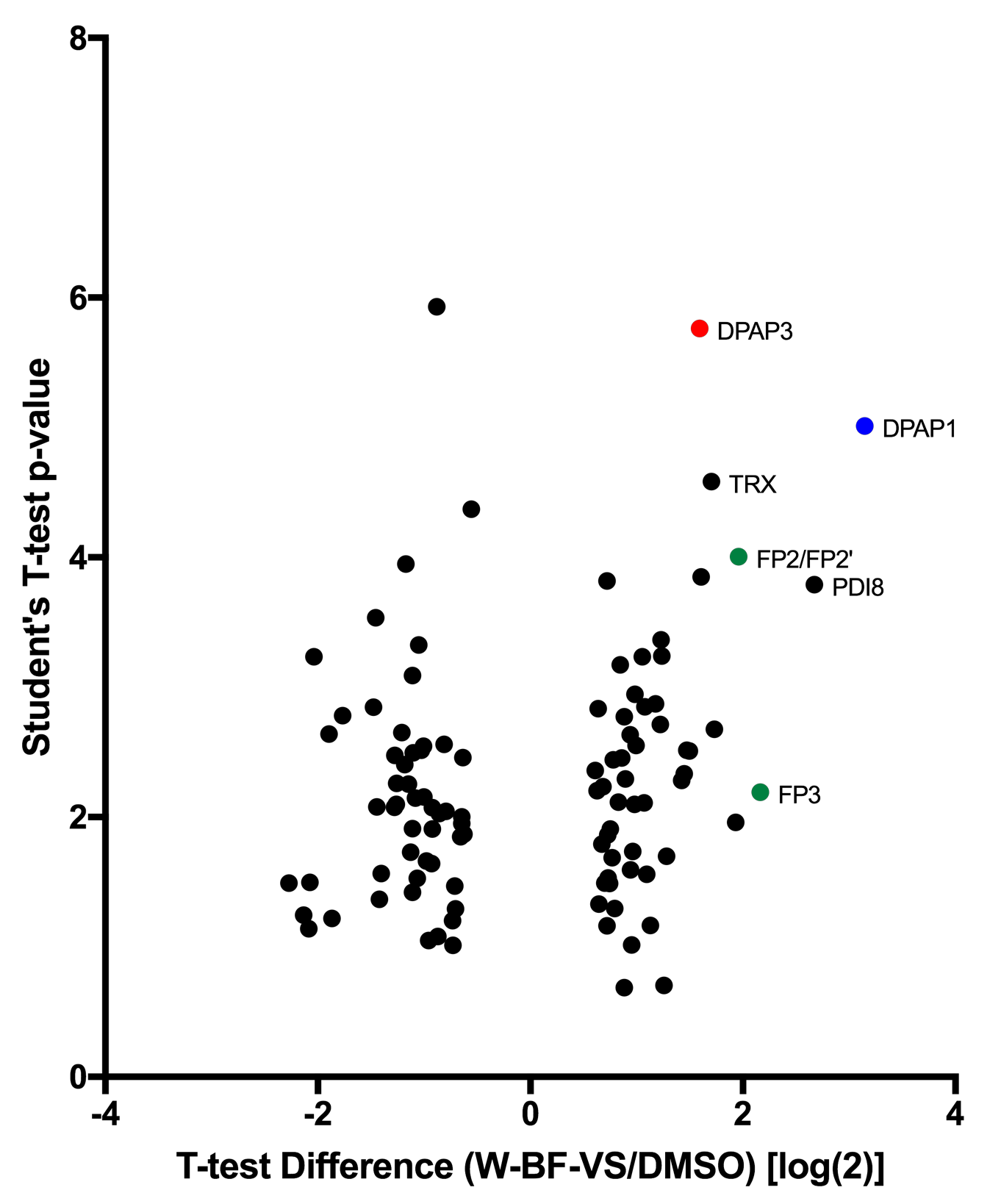

Supplement: S2 Fig — Volcano plot showing enrichment of clan CA proteases and enzymes containing reactive cysteine when schizont lysates were treated with W-BF-VS versus DMSO. (TIFF) [file pone.0227341.s002.tiff]

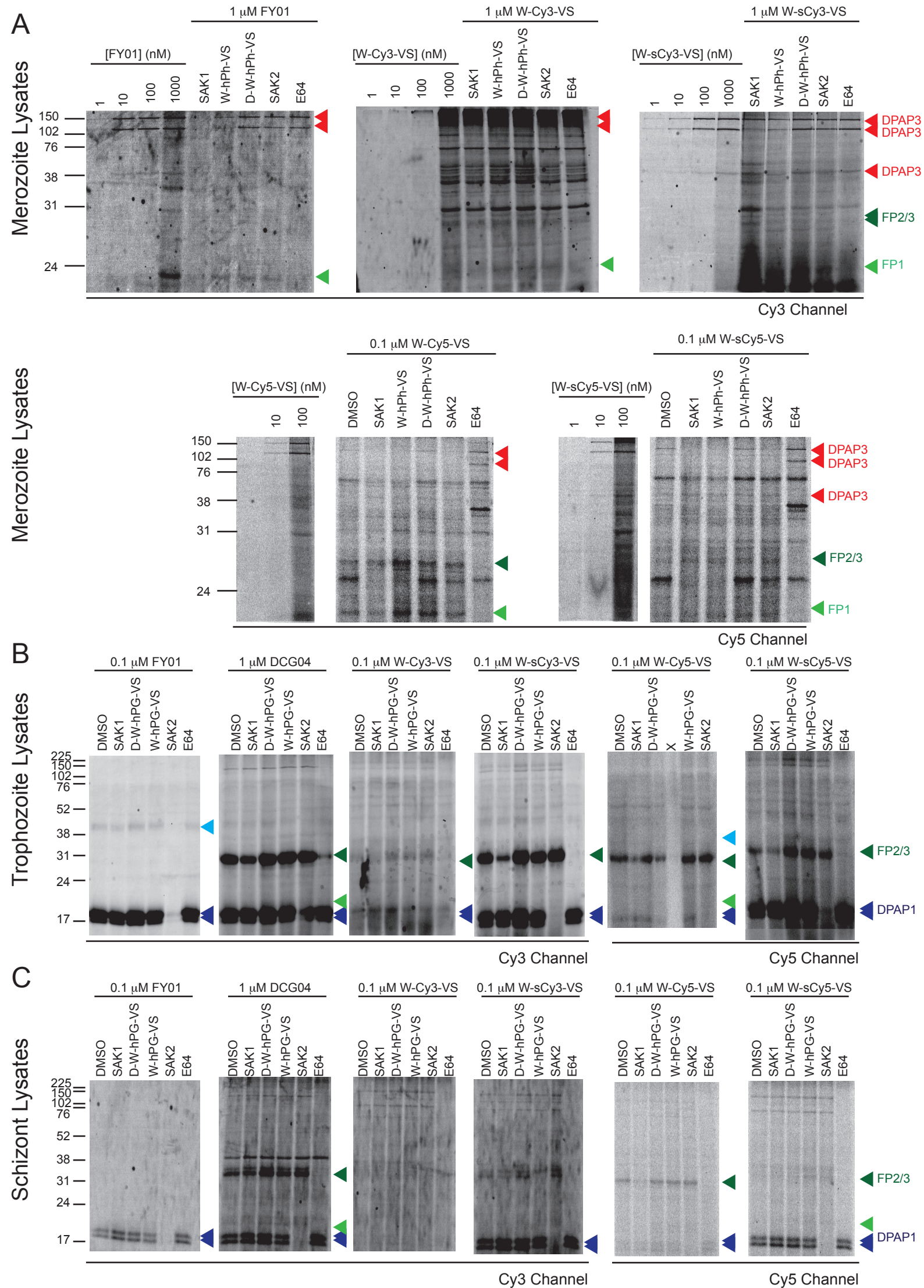

Supplement: S3 Fig — (A) Merozoite lysates diluted 1:10 in acetate buffer were treated for 1 h with 1–1000 nM of the indicated ABPs. For the highest ABP concentration, samples were also pre-treated for 30 min with 10 μM of SAK1, W-hPG-VS D-W-hPG-VS, SAK2 or E64. (B-C) Lysates collected at trophozoite (B) or schizont (C) stages were diluted in acetate buffer (pH 5.5), pre-treated for 30 min with DMSO or 10 μM of different known covalent inhibitors of DPAP1 (SAK2), DPAP3 (SAK1 or W-hPG-VS), the FPs (E64), or the negative control compound D-W-hPG-VS. This was followed by 1 h labelling with the different ABPs at 0.1 μM except for DCG04 that was used at 1 μM concentration. (A-C) The fluorescent bands corresponding to DPAP1, DPAP3, FP1, and FP2/3 are indicated by blue, red, light green, and dark green arrowheads, respectively. Each page represent a different biological replicate. (PDF) [file pone.0227341.s003.pdf]

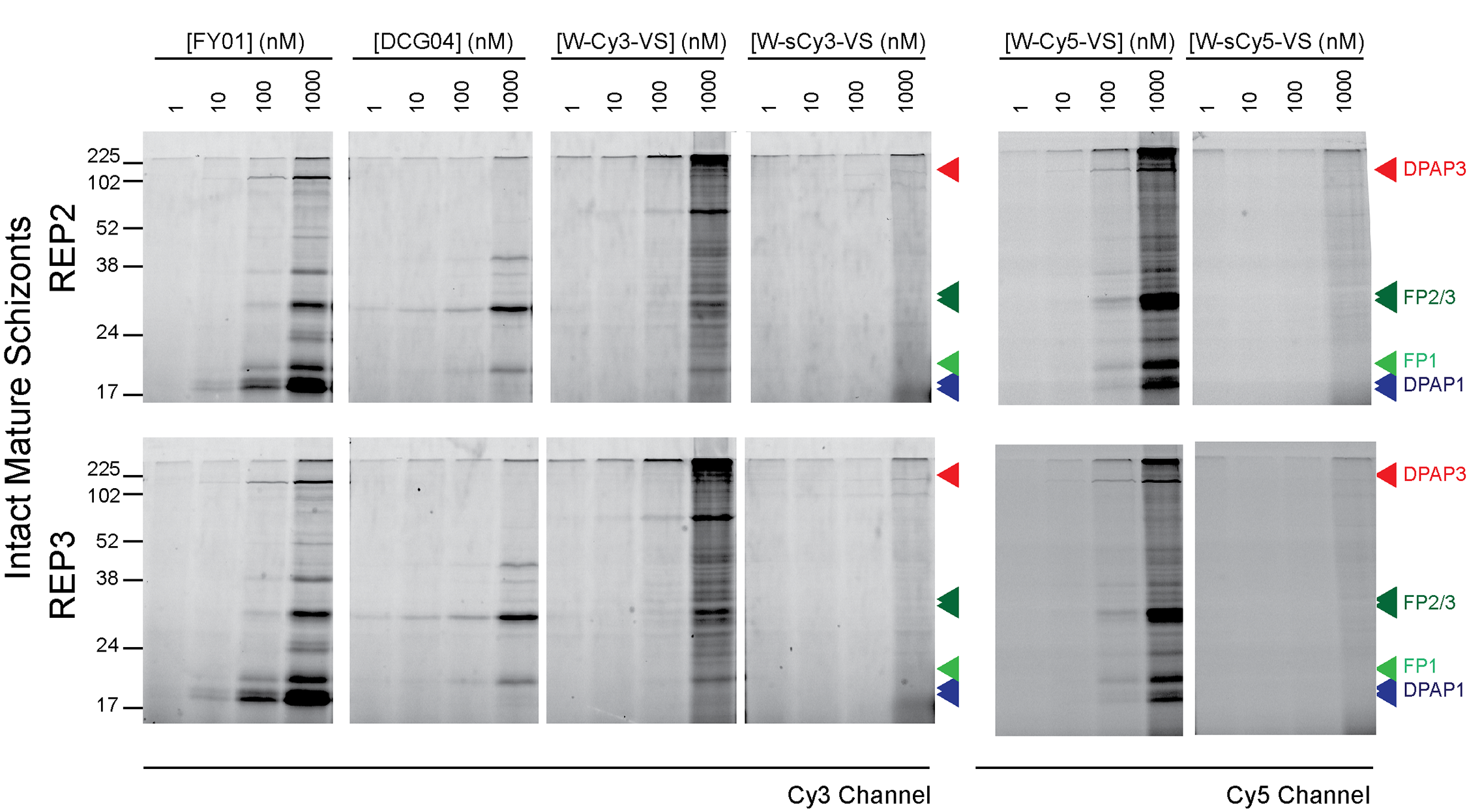

Supplement: S4 Fig — Very mature schizonts were diluted ten-fold in RPMI and treated with different concentrations of probes for 1 h. Samples were run on a SDS-PAGE gel, and the labelled proteins detected using at fluorescence scanner. Bands corresponding to DPAP1, DPAP3, FP1, and FP2/3 are indicated by blue, red, light green, and dark green arrowheads, respectively. Top and bottom panels represent different biological replicates. (TIF) [file pone.0227341.s004.tif]

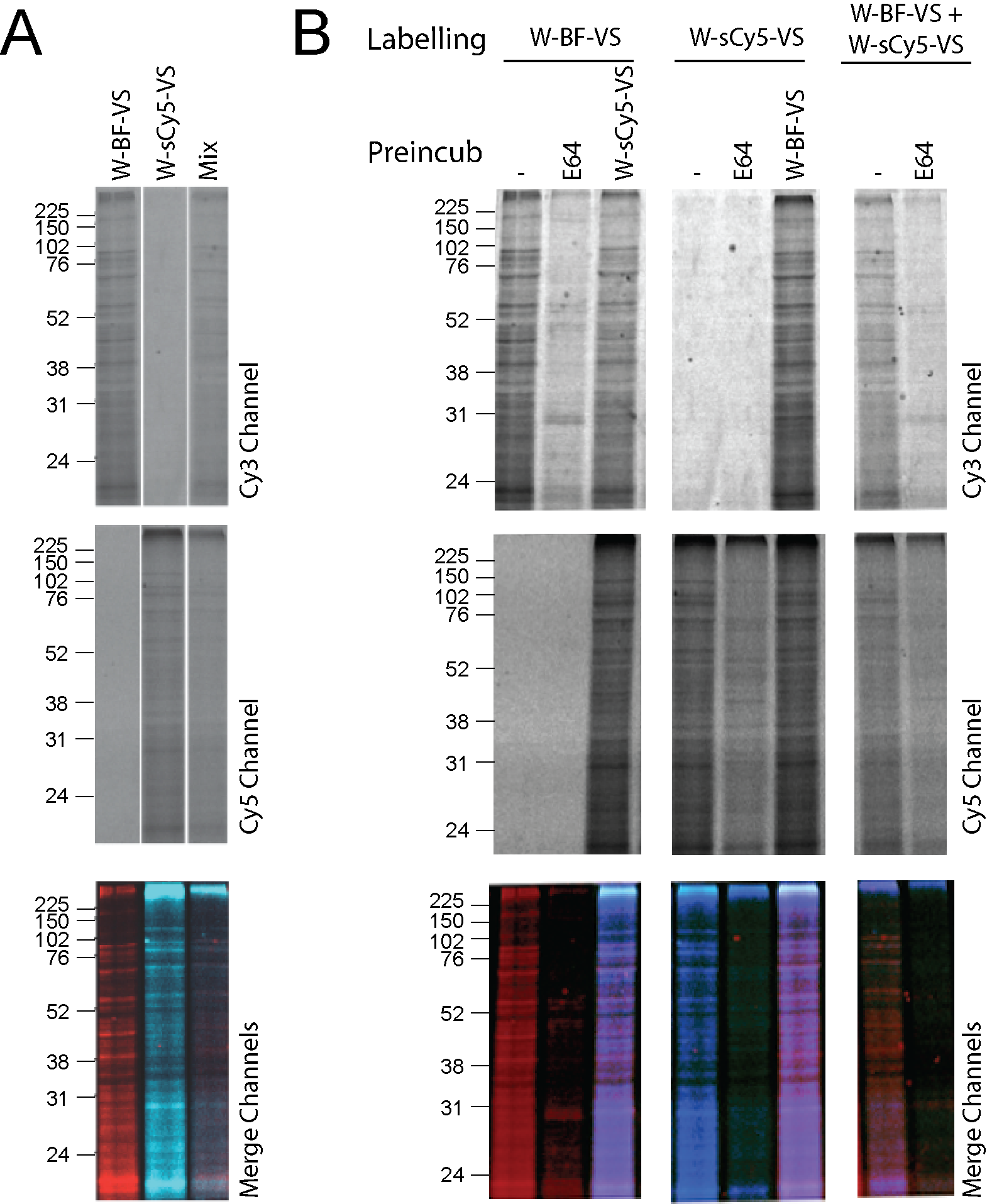

Supplement: S5 Fig — (A) Schizont lysates were treated for 1 h either with 0.5 μM of W-sCy5-VS, W-BF-VS, or a mixture of both probes, each at 0.5 μM (Mix). (B) Schizont lysates were pre-treated for 30 min with either 10 μM of E64 or 0.5 μM of one of the probes followed by 1h labelling with the other probe or a combination of both, each at 0.5 μM (last two lanes). After running the samples in a SDS-PAGE gel, the gel was scanned either in the Cy5 and Cy3 channels. The composite image (merge channels images) shows very similar labelling profiles for both probes and a clear co-migration of the labelled bands in the Mix sample. Note that E64 is able to outcompete probe labelling for most of the bands. While pre-treatment with W-sCy5-VS results in a decrease in the labelling intensity of the bands labelled by W-BF-VS, pre-treatment with W-BF-VS does not decrease the labelling of band by W-sCy5-VS. This is consistent with W-sCy5-VS being a more potent and reactive probe. (TIF) [file pone.0227341.s005.tif]

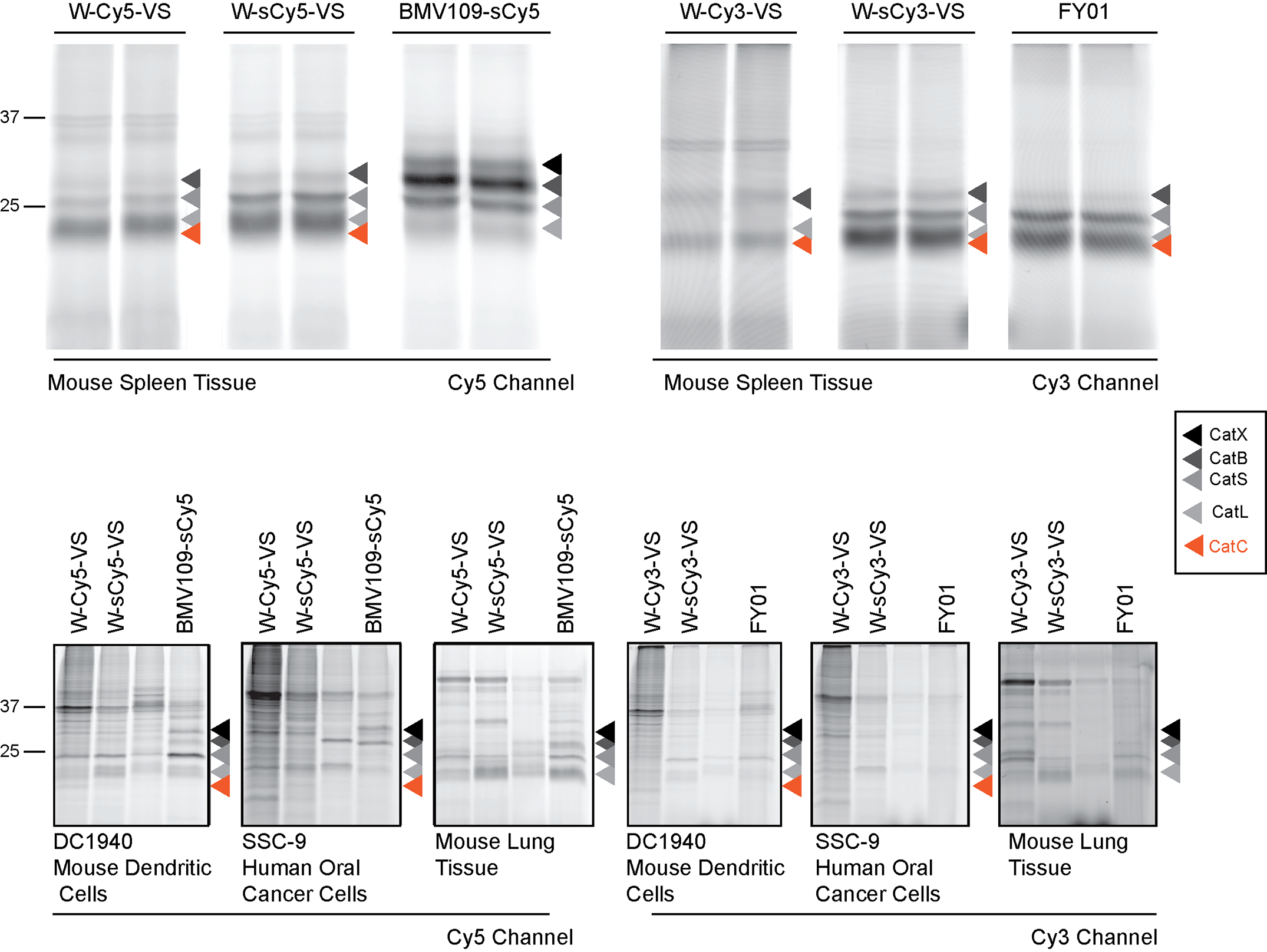

Supplement: S6 Fig — Lysates from the indicated cell lines or from mouse lung or spleen tissues were treated with 1 μM of the different ABPs for 30 min. The identity of the different cysteine cathepsins are indicated with different coloured arrowheads. (TIF) [file pone.0227341.s006.tif]
